# Machine Learning for Differentiating Essential Tremor: A Scoping Review

**DOI:** 10.5334/tohm.1182

**Published:** 2026-05-06

**Authors:** David M. Fletcher, Kaitlyn E. Heintzelman, Sumesh B. Ramasamy, Allison Marks, Joseph C. Melott, Amy W. Amara, Adeel A. Memon

**Affiliations:** 1 School of Medicine, West Virginia University, Morgantown, WV, 26505, USA; 2 Department of Neuroscience, West Virginia University, Morgantown, WV, 26505, USA; 3 Department of Chemical and Biomedical Engineering, West Virginia University, Morgantown, WV, 26505, USA; 4 Department of Computer Science and Electrical Engineering, West Virginia University, Morgantown, WV, 26505, USA; 5 Department of Biological Sciences, West Virginia University, Morgantown, WV, 26505, USA; 6 Department of Neurology, West Virginia University, Morgantown, WV, 26065, USA; 7 Department of Neurology, University of Colorado, Anschutz Medical Campus, Aurora, CO, 80045, USA; 8 Department of Neurology, West Virginia University, Morgantown, WV, 26505, USA

**Keywords:** Scoping Review, Essential Tremor, Machine Learning, Artificial Intelligence, Movement Disorders

## Abstract

**Background:**

Essential tremor (ET) is the most common movement disorder, affecting ~6% of adults over 65 [1]. Differentiating ET from other tremors remains clinically challenging due to overlapping features and variable presentation. Artificial intelligence (AI), particularly machine learning (ML), has emerged a potential tool to support neurologists by enhancing pattern recognition and complementing traditional assessments in complex cases. This is the first scoping review examining ML’s potential role in distinguishing essential tremor from other tremor types.

**Methods:**

A systematic, scoping search was conducted using PubMed, Cochrane, and Scopus through April 2025, in accordance with PRISMA guidelines-ScR [2]. Studies applying AI to distinguish ET from other tremors were included. Of 548 studies screened, 97 underwent full-text review, with data extracted from 46.

**Results:**

46 included studies encompassed 6,051 patients, including 2,358 with ET. ML models utilized diverse inputs: accelerometers, gyroscopes, voice recordings, Archimedes spirals, EMG, and video. Common algorithms were included vector machines (18 articles), k-nearest neighbors (9 articles), and convolutional neural networks (8 articles). There was a high amount of heterogeneity in reporting data, severely limiting between study comparisons. Reported classification accuracies ranged from 60% to 100% (mean: 89%). However, heterogeneity in data types, methodologies, and reporting limited cross-study comparability.

**Conclusions:**

ML shows promise as a decision-support tool by recognizing tremor features that may complement, but not replace, expert clinical assessment, particularly in diagnostically ambiguous cases. To enable clinical adoption, future studies must address current heterogeneity, develop standardized datasets, implement automated preprocessing, and focus on clinically feasible data sources.

Tremor is a common yet diagnostically challenging neurological symptom spanning a range of disorders such as essential tremor (ET), Parkinson’s Disease (PD), dystonic tremor (DT), and functional tremor (FT) [3,4]. ET is the most prevalent, with an estimated global incidence of 13.3 per 1,000 individuals, rising to 57.9 per 1,000 in those over 65 years of age [1,5]. Despite its frequency, differentiating ET from other tremor types remains difficult due to overlapping clinical features, heterogeneous patient presentations, and variability in clinical interpretation [6]. Diagnosis typically relies on clinical examination with possible incorporation of a combination of structured tools, including tremor rating scales, Archimedes spiral analyses, and electromyography (EMG); however, these tools ultimately depend on pattern recognition [7,8,9]. As clinical data grows in volume and complexity, there is a growing need for tools, such as AI, that can support neurologists by streamlining this pattern-based decision-making. Importantly, ML-based approaches in this field are not intended to establish a biological ground truth for ET, which remains a clinical diagnosis, but rather to model and support clinician-based pattern recognition.

Machine learning (ML) offers a promising avenue for augmenting clinical diagnosis by supporting the pattern recognition that neurologists routinely apply in clinical practice [10,11]. As tremor assessment increasingly involves multimodal data such as accelerometry, EMG, or handwriting samples, ML models can assist in identifying subtle phenotypic differences that may complement traditional evaluation [11,12,13]. A range of approaches, including supervised and unsupervised learning as well as ensemble classifiers have been used to analyze these complex datasets and extract diagnostic features [11,12,13]. Beyond neurology, ML has already led to major breakthroughs in biomedical science, exemplified by AlphaFold’s success in protein structure prediction [14], and is actively being explored to assist in early disease detection, such as in breast cancer screening and diabetic retinopathy [15,16]. However, despite its potential, ML remains underutilized in tremor classification, particularly in differentiating ET from PD, DT, or FT, highlighting a gap between technical innovation and clinical implementation.

Recognizing the increasing need for objective methods to aid neurologists in their decision-making [17,18], this scoping review offers a timely first step toward addressing the ongoing diagnostic challenges posed by ET [3,11]. Given the substantial heterogeneity in study designs, patient populations, and methodologies, a scoping review approach was chosen to map the current literature and identify research gaps, rather than conduct a systematic review and meta-analysis. Due to the growing interest in leveraging AI for movement disorder diagnosis, this scoping review seeks to map and synthesize current research on the use of AI methods to distinguish ET from other tremor disorders. While previous reviews have explored machine learning approaches to tremor classification [18], important differences exist in scope and focus. In particular, the review by De et al. (2023) primarily addressed PD and emphasized accelerometer-based tremor assessment, with a focus on comparing machine learning methodologies [18]. In contrast, the present scoping review specifically targets ET and its differentiation from other tremor disorders. Furthermore, unlike prior work, we do not restrict inclusion based on data acquisition modality, thereby capturing studies using both wearable sensor-based and non-wearable clinical or instrumental data sources. This broader inclusion strategy allows for a more comprehensive mapping of AI/ML approaches applied to essential tremor across diverse datasets and methodological frameworks.

As a result, we examine the diversity of computational approaches employed, the types of clinical and sensor-based data analyzed, and the diagnostic performance achieved. In doing so, we aim to provide a comprehensive overview of the state of the field, identify methodological limitations, and outline opportunities for future development and validation of AI-assisted tremor classification tools. This synthesis highlights substantial heterogeneity across studies in three key domains: patient populations, tremor data, and AI techniques/data reporting, underscoring the need for a structured overview. By identifying methodological strengths, limitations, and evidence gaps, this review aims to inform future research priorities and facilitate clinical integration of AI through initiatives such as an expert–led consortium, ultimately improving differential diagnosis and access to high-quality care.

## Methods

This scoping review was conducted in accordance with the 2020 PRISMA-ScR guidelines [2]. All studies identified for potential inclusion were indexed from PubMed, Cochrane Library, or Scopus. For each database, all articles published up to the date of data curation (April 2025) were included. The search strategy consisted of two parts: tremor and AI. Given the wide range of possible patient data types, the search was intentionally inclusive and did not restrict the type of data collected from the patient. Briefly, the types of tremor search consisted of medical subject headings (MeSH) of “Tremor” and “Essential Tremor.” The AI string contained terms related to machine learning and artificial intelligence including: “Machine Learning,” “Supervised Machine Learning,” “Unsupervised Learning,” and “Deep Learning.” These two strings were then combined using the AND boolean operator, which retrieves records containing both terms, according to the specific syntax requirements of each database. All search strategies used, along with subsequent article hits, can be found in the supplemental data S1.

Search strings yielded 548 studies, which were then uploaded to Covidence (Melbourne, Australia). Covidence is an online software that facilitates the screening of potential articles for scoping or systematic reviews; the software contains an artificial intelligence-based learning system that automatically removes duplicates and learns from the reviewers’ choices to remove irrelevant articles.

Eligible articles marked for inclusion in this scoping review included all published articles that used AI to distinguish ET from other tremors. Abstracts, commentaries, or posters were not included for analysis. Similarly, any article that only included animal studies, was not published (peer-reviewed), or was not written in English was excluded. Articles that used AI for different purposes were excluded. Additionally, studies that only compared ET to healthy controls or focused primarily on differentiating non-ET tremor types were excluded, as our scope was intentionally limited to studies distinguishing ET from at least one other tremor disorder. Review articles were not included in the final study count; however, the team reviewed them to identify any additional articles not detected by the search terms. No such additional articles were identified.

To assess article eligibility, two authors (DMF and KEH) initially screened each article based on the title and abstract. This screening used defined PICO criteria described briefly above and expanded in supplemental data S2. Both authors had to agree on article inclusion in order for it to proceed to a second review. If there was a conflict, a third, neutral author (SBR) made the final decision on inclusion. After the first review, included articles were reviewed for a second time, where the first two authors read the full text and decided on inclusion based on the PICO criteria. Again, the third author determined inclusion for any articles with disagreements between the original two authors.

If an article passed two rounds of screening, the authors then extracted data. Data extracted from each article included: study design, total number of participants (for each tremor type), the data recorded, the AI method implemented, and the resulting accuracy, sensitivity, and specificity.

The Joanna Briggs Institute (JBI) Critical Appraisal Tool was used to report bias for each paper [19]. The JBI Critical Appraisal Tool classifies papers based on their design (i.e., case report, clinical trial, cohort study, etc.), and reviewers score the risk of bias using a series of 8–11 questions [19]. Two reviewers (DMF and KEH) independently scored each paper. If there were conflicts, a third reviewer (SBR) broke the tie. The scores were then used to develop a bias assessment, which consisted of a scale with options of “yes, no, or unclear” for each specific question. The overall bias assessment for each study was an average of the questions that scored a “yes.”

## Results

**Figure 1:**
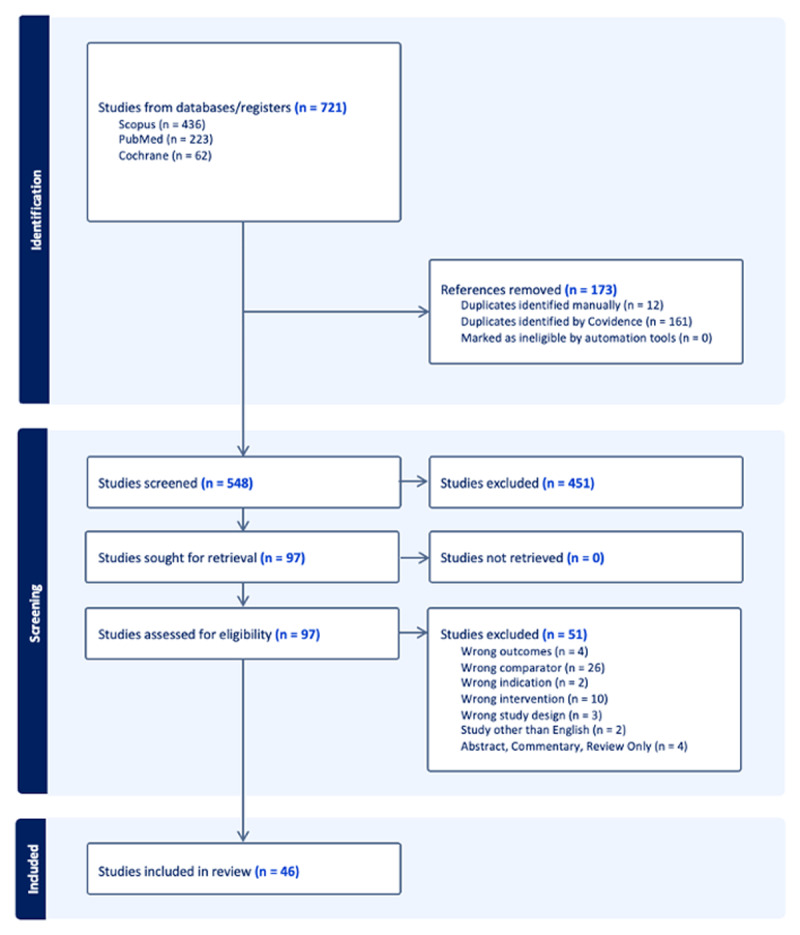
PRISMA Figure. This shows the outline of included studies as well as the reason for exclusion.

The process for manuscript selection is displayed in Figure 1. A total of 721 articles were identified in the literature review. Covidence automatically removed 161 duplicates, and 12 additional duplicates were identified manually, leaving 548 articles for screening. Two reviewers independently assessed the deduplicated titles and abstracts from the literature search for relevance, which resulted in the exclusion of 451 additional articles. Subsequently, the 97 articles whose titles and abstracts met the initial inclusion criteria underwent a full-text review to evaluate study design quality and determine which met the final inclusion criteria, resulting in 46 included articles [11,20,21,22,23,24,25,26,27,28,29,30,31,32,33,34,35,36,37,38,39,40,41,42,43,44,45,46,47,48,49,50,51,52,53,54,55,56,57,58,59,60,61,62,63,64]. This process, including the rationale for article exclusion post-full-text review, is detailed in Figure 1. The final set of studies included articles published between 1999 and 2025, reflecting a quarter of a century of using AI to differentiate ET from other types of tremors. Supplemental data S3 presents each article included in this scoping review, grouped by its study design. Table 1 contains the information below in one central format.

**Table 1:** Comprehensive table of included studies, number of patients, tremor types, data acquisition types, types of AI/ML, and diagnostic testing measures.


STUDY NAME	TOTAL N	TREMOR TYPES AND N	DATA TYPES	AI METHODS	ACCURACY/SENSITIVITY/SPECIFICITY

Ai et al. 2007	26	ET: 6 PD: 10 PT: 10	Hand Accelerometer	BPNN	Accuracy: 92.9%

Ai et al. 2008	40	ET: 10 PD: 15 PT: 15	Hand Accelerometer	BPNN	Accuracy: 96.67%

Ai et al. 2011	25	ET: 10 PD: 15	Hand Accelerometer	SVM	Accuracy: 98% Sensitivity: 97.5% Specificity: 98.33%

Anandapadmanabhan et al. 2025	1077	ET: 215 PD: 211 ET+: 208 DT: 365 CA: 78	Archimedes’ Spirals (pen/paper)	CNN	Accuracy: 81%

Aracri et al. 2024	72	ET: 32 PD: 40	EMG	GB/xGB	Accuracy: 84.5% Sensitivity: 85% Specificity: 83.8%

Aubin et al. 2012	14	ET: 7 PD: 7	Inertial Measurement Unit	SVM	Accuracy: 85.7%

Balachandar et al. 2022	105	ET: 49 PD: 30 DT: 26	Hand Accelerometer (smartphone), Tremor Rating Scale	UMAP; LOOCV	Accuracy: 88%

Chandra Reddy et al. 2024	21	ET: 9 PD: 5 CT: 7	Video	CNN	Accuracy: 87.5%

Darnall et al. 2012	10	ET: 3 PD: 6 ET/PD: 1	Gyroscope, Archimedes’ Spirals (Digital), Tremor Rating Scale	DT, kNN, MP, NB, RF, SVM	Accuracy: 82%

Duque et al. 2022	39	ET: 20 PD: 19	Gyroscope (smartphone)	kNN, SVM	Accuracy: 77.8% Sensitivity: 75.7% Specificity: 80%

Engin et al. 2007	12	ET: 3 PD: 9	Hand Accelerometer	ANN	Accuracy: 91.02% Sensitivity: 92.97% Specificity: 87.13%

Ferreira et al. 2022	37	ET: 10 PD: 27	Hand Accelerometer, Gyroscope	DT, kNN, NB, RF, SVM	Accuracy: 100%

Ghassemi et al. 2016	24	ET: 11 PD: 13	Hand Accelerometer, EMG	SVM	Accuracy: 83%

Gonzalez et al. 2014	154	ET: 34 PD: 120	Hand position, velocity, acceleration	MP	Accuracy: 80%

Groznik et al. 2013	132	ET: 52 PD: 46 Mixed: 34	Archimedes’ Spirals (Digital)	ABML	Accuracy: 91% Sensitivity: 86% Specificity: 90%

Hossen et al. 2012	40	ET: 20 PD: 20	Hand Accelerometer, EMG	BPNN	Accuracy: 91.6% Sensitivity: 95% Specificity: 88.2%

Hossen et al. 2022	40	ET: 20 PD: 20	Hand Accelerometer, EMG	FF-BPNN	Accuracy: 92.5% Sensitivity: 98.47% Specificity: 100%

Ishii et al. 2020	50	ET: 24 CA: 26	Archimedes’ Spirals (Pen/Paper)	CNN	Accuracy: 70% Sensitivity: 44% Specificity: 79%

Jakubowski et al. 2002	174	ET: 35 PD: 39 PT: 100	Hand Accelerometer	MP	Accuracy: 97%

Kovalenko et al. 202	57	ET: 13 PD: 42 Other: 2	Video	GPC, GB/xGB, LR, RF, SVM	Accuracy: 77%

Lee et al. 2023	105	ET: 18 PD: 87	Video, Tremor Rating Scale	LSTM	Accuracy: 60% Sensitivity: 60% Specificity: 95%

Li et al. 2023	31	ET: 12 PD: 19	Hand Accelerometer	SVM; LOOCV	Accuracy: 100% Sensitivity: 100% Specificity: 100%

Lin et al. 2023	164	ET: 80 PD: 84	Inertial Measurement Unit (wearable), Task	LR; LOOCV	Accuracy: 84% Sensitivity: 85.9% Specificity: 82.1%

Locatelli et al. 2020	24	ET: 7 PD: 17	Hand Accelerometer	DT, DA, kNN, NB, SVM	Accuracy: 90.9% Sensitivity: 94.1% Specificity: 80%

Moon et al. 2020	567	ET: 43 PD: 524	Hand Accelerometer, Gyroscope (wearables)	DT, GB/xGB, kNN, LR, RF, SVM	Accuracy: 89% Sensitivity: 61%

Nanayakkara et al. 2025	66	ET: 15 PD: 51	Hand Accelerometer	LSTM	Accuracy: 95%

Nanda et al. 2015	2	ET: 1 PD: 1	Hand Accelerometer, EMG	FF-BPNN	NA

Oktay et al. 2020	40	ET: 17 PD: 23	Video	LSTM	Accuracy: 90%

Piepjohn et al. 2022	478	ET: 305 PD: 173	Hand Accelerometer, EMG	CNN, DT	Accuracy: 85.76%

Ranjan et al. 2020	27	ET: 13 PD: 14	Hand Accelerometer	kNN, RF, SVM; GMM, kM	Accuracy: 94.68% Specificity: 98.73%

Saad et al. 2024	43	ET: 14 PD: 29	Video	GB/xGB, SVM	Accuracy: 93% Specificity: 96%

Sanderson 2020	46	ET: 12 PD: 34	Inertial Measurement Unit	SVM	Accuracy: 92.4%

Seedat 2020	1039	ET: 669 PD: 370	Archimedes’ Spirals (Pen/Paper)	CNN	Accuracy: 92%

Shahtalebi 2020	162	ET: 81 PD: 81	Hand Accelerometer	PHTnet	NA

Shahtalebi 2021	162	ET: 81 PD: 81	Hand Accelerometer	DA	Accuracy: 95.5%

Skaramagkas 2020	15	ET: 3 PD: 12	Hand Accelerometer	DT, DA, EL, kNN, SVM	Accuracy: 100%

Skaramagkas 2021	15	ET: 3 PD: 12	Hand Accelerometer	DT, DA, EL, kNN, SVM	Accuracy: 100%

Spyers-Ashby 1999	57	ET: 21 PD: 19 MS: 17	Hand Accelerometer	kNN	Accuracy: 60%

Surangsrirat 2016	52	ET: 20 PD: 32	Gyroscope	SVM	Accuracy: 100% Sensitivity: 100% Specificity: 100%

Tang 2024	140	ET: 53 PD: 87	Hand Accelerometer, EMG	CAMN	Accuracy: 97.18%

Tavakkoli 2014	40	ET: 20 PD: 20	EMG	CNN, SVM	Accuracy: 95.75%

Teo 2024	143	ET: 25 PD: 118	Task	LSTM	Accuracy: 90% Sensitivity: 70% Specificity: 100%

Vescio 2023	40	ET: 20 PD: 20	Inertial Measurement Unit (wearables)	GB/xGB, RF	Accuracy: 92% Sensitivity: 96% Specificity: 87%

Weede 2024	339	ET: 209 PD: 130	Hand Accelerometer	CNN	Accuracy: 88.12%

Xing 2022	79	ET: 28 PD: 51	Hand Accelerometer, EMG	BPNN, CNN, GB/xGB, LR, RF, RRC, SVM	Accuracy: 85% Specificity: 64%

Yang 2020	26	ET: 5 PD: 21	Archimedes’ Spirals (Digital)	GRNN	Accuracy: 88.27%


**Tremor Types: CA**–Cerebellar Ataxia; **CT**–Cerebellar Tremor; **DT**–Dystonic Tremor; **ET**–Essential Tremor; **ET+**–Essential Tremor Plus; **ET/PD**–Essential Tremor/Parkinson’s Disease; **Mixed**–Mixed Tremor; **MS**–Multiple Sclerosis; **Other**–Other tremor, not otherwise classified; **PD**–Parkinson’s Disease; **PT**–Physiological Tremor. **Data Types: EMG** – Electromyography; **Inertial Measurement Unit** – combines accelerometer, gyroscope, and magnetometer data in one device. **AI Types: Supervised Learning: ANN**–Artificial Neural Network; **BPNN**–Back Propagation Neural Network; **CAMN**–Cross Attention Mechanism Network; **CNN**–Convolutional Neural Network; **DT**–Decision Tree; **DA**–Discriminate Analysis; **EL**–Ensemble Learning; **FF-BPNN**–Feed Forward Back Propagation Neural Network; **GPC**–Gaussian Process Classifier; **GRNN**–Generalized Regression Neural Network; **GB/xGB**–Gradient Boosting/eXtreme Gradient Boosting; **kNN**–k Nearest Neighbors; **LR**–Logistic Regression; **LSTM**–Long Short Term Memory; **MP**–Multilayer Perceptron; **NB**–Naive Bayes; **RF**–Random Forest; **RRC**–Ridge Regression for Classification; **SVM**–Support Vector Machine. **Unsupervised Learning: GMM**–Gaussian Mixture Model; **kM**–k Means. **Semi-Supervised Learning: PHTnet**–Patch-wise Hierarchical Transformer Network; **UMAP**–Uniform Manifold Approximation and Projection for Dimension Reduction. **Multiple: ABML**–Argument-Based Machine Learning. **Validation: LOOCV**–Leave One Out Cross Validation.

### Study Types and Quality

No prospective studies met the inclusion criteria for this scoping review. All 46 articles best fit the classification of “Diagnostic test accuracy studies” from the JBI classification system. The average study quality was 83.91%. Complete bias assessment scores for each study can be found in the supplemental data S4.

### Types of Tremor

A total of 6,051 patients were included across the 46 articles. The tremor types represented include ET, PD, physiological tremor, DT, cerebellar tremor, mixed PD and ET, essential tremor plus, cerebellar ataxia, and multiple sclerosis. The most common type of tremor differentiated from ET was PD, examined in 45 articles. This was followed by physiological tremor (2 articles), DT (2 articles), mixed PD and ET (2 articles), and cerebellar ataxia (2 articles). Supplemental data S5 summarizes the number of patients associated with each tremor type and the corresponding number of studies that distinguish them from ET.

### Types of Data Acquired and AI Models

**Figure 2:**
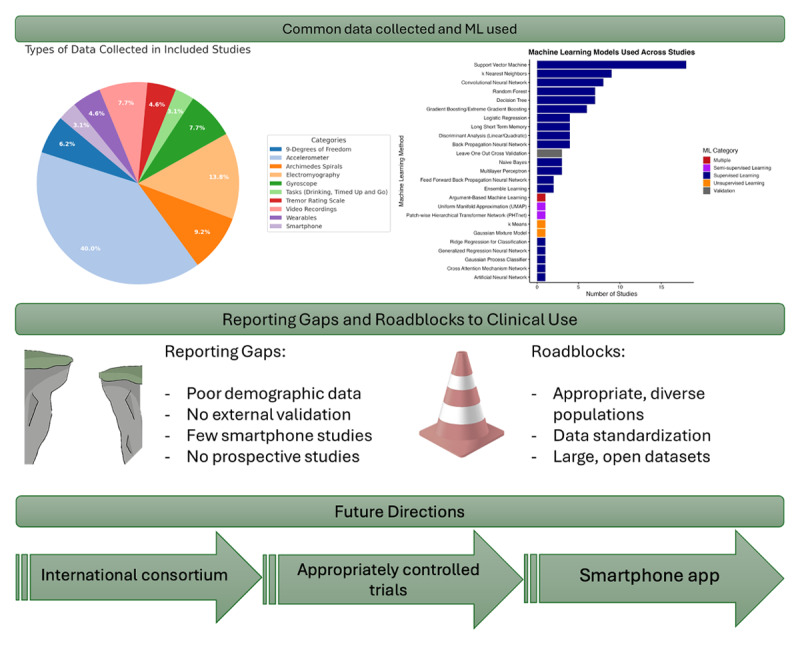
Conceptual overview of the current state and future directions for machine learning in tremor classification. Key future steps include conducting rigorous clinical trials, aggregating data into publicly available datasets, and developing an international, consortium-led smartphone application.

From each included article, both the type of data acquired from participants and the AI models employed were recorded. Common data types included accelerometer data (26 articles), EMG (9 articles), Archimedes spirals (6 articles), gyroscope data (5 articles), and video recordings (5 articles). A comprehensive list of all recorded patient data types is provided in supplemental data S6. The AI models used varied across studies, with the most frequently applied being support vector machines (18 articles), k-nearest neighbors (9 articles), and convolutional neural networks (8 articles) (Figure 2). A comprehensive list of AI models used, along with their respective classifications, is presented in supplemental data S7.

### Reported Accuracy, Sensitivity, and Specificity of Models

Of the 46 included articles, 44 reported model accuracy. Due to a large amount of heterogeneity in reporting, patient characteristics, and AI models used, the following data cannot be compared directly. Reported classification accuracies ranged from 60% to 100%; however, these values must be interpreted cautiously, as they reflect agreement with clinician-labeled diagnoses in the setting of known diagnostic uncertainty and heterogeneous study designs. Sensitivity and specificity were reported in 16 and 18 articles, respectively. Sensitivity ranged from 0.44 to 1.00, and specificity ranged from 0.64 to 1.00. Additionally, overall sensitivity and specificity were calculated from 15 articles that provided sufficient data to extrapolate true positives, false positives, true negatives, and false negatives. These values were pooled across studies, yielding an overall sensitivity of 0.83 and an overall specificity of 0.91. As stated, due to methodological variability across studies, including differences in data types and evaluation criteria, no definitive conclusions could be drawn about which model performed best or worse.

## Discussion

This scoping review evaluated 46 studies that utilized ML models with electrophysiological signals, kinematic measurements, drawing tasks, functional assessments, clinical rating scales, and video recordings to distinguish ET from other tremor types across 6,501 patients. All studies met the JBI classification for diagnostic test accuracy studies, with an average quality score of 83.91%. The included studies employed a variety of ML approaches, with reported classification accuracies ranging from 60% to 100% and an overall average of 89%. However, there was substantial heterogeneity in patient demographic reporting, tremor data types, AI models used, and data reporting methods. This variability prevented direct comparison across studies and precluded systematic review or meta-analysis techniques. Importantly, given the clinical overlap between tremor syndromes, these findings highlight the potential of ML models as pattern recognition tools that can support and complement neurologists’ clinical expertise in the assessment of tremor disorders.

Examination of the patient populations and study designs revealed limitations in demographic reporting and study methodology. Across the 46 studies, reporting of basic patient demographic information such as race, gender, and age was inconsistent, limiting comparisons across cohorts. Furthermore, no study included case-matched controls, nor were any randomized controlled trials identified. These design elements are important for objectively evaluating AI performance in distinguishing ET from other tremor types. The emergence of the ET-plus classification introduces additional complexity for ML-based tremor studies. Because ET-plus is variably defined, inconsistently applied across centers, and often underreported, its presence likely contributes to diagnostic heterogeneity and label noise in existing datasets [3,6]. Addressing these limitations in future research would allow more robust comparisons across AI models and study settings.

Analysis of data acquisition methods across the 46 included studies revealed clear trends in the modalities used for ML model input. Accelerometer data was the most common modality, appearing in 26 studies, followed by EMG (9 articles), Archimedes spirals (6 articles), gyroscope data (5 articles), and video recordings (5 articles). From a practical standpoint, however, not all data acquisition methods translate easily into clinical practice. For example, while EMG provides rich electrophysiological detail and often incorporates accelerometer sensors, full EMG-based assessment workflows may be difficult to implement routinely in outpatient settings due to time requirements, technical expertise, reimbursement constraints, and patient tolerance [65,66]. In contrast, Archimedes’ spiral drawings, which were used in only six studies, are widely adopted in clinical assessment because of their simplicity, minimal equipment requirements, and ability to visually capture tremor amplitude and variability [67,68,69]. Although accelerometer and EMG-based approaches dominate the research space, they may be less feasible in typical outpatient neurology settings, highlighting a disconnect between research priorities and clinical practice. A potential middle ground may lie in smartphone-based assessments that integrate accelerometer and gyroscope data. Although only two studies utilized smartphones, they reported accuracies of 77.90% and 88.00%, comparable to more complex sensor configurations but with far greater theoretical ease of deployment [26,29].

In addition to the range of data types collected, the ML techniques used to distinguish ET from other tremor types also varied considerably. The most common approach was support vector machines (SVM), used in 18 studies, followed by k-nearest neighbors (KNN) in nine studies and convolutional neural networks (CNN) in eight studies. These are all supervised learning models that rely on labeled examples during training to classify new data. For instance, if the algorithm is trained using tremor patterns labeled as PD or ET, it learns decision boundaries that allow it to distinguish between these categories [18]. However, model training depends heavily on the availability and quality of labeled datasets, an area where the field still faces major limitations. Most studies relied on small, institution-specific datasets, underscoring the need for large, standardized, publicly available datasets. A consortium-led initiative would be well positioned to coordinate this effort and develop equitable AI models that could aid diagnosis or potentially streamline clinical visits for future patients [70].

While standardizing and simplifying data inputs is essential for clinical implementation, evaluating how different ML models perform is equally important, though not straightforward. Comparing diagnostic accuracy across studies proved challenging because of substantial methodological variability. Each study used its own combination of data types, preprocessing steps, model architectures, and evaluation criteria. In addition, many studies employed ensemble or hybrid approaches, making it difficult to isolate the contribution of individual ML methods. Reporting of evaluation metrics also varied; although most studies reported overall accuracy, fewer included sensitivity, specificity, or area under the curve, which are important for assessing clinical utility. Moreover, almost all models were trained and validated on internal datasets from single institutions, with little use of external validation cohorts. This limits understanding of model generalizability across diverse populations and clinical settings.

Among commonly used models, SVMs appeared in 18 studies, with reported accuracies ranging from 83% to 100%. KNN was used in nine studies, with accuracies ranging from 60% to 94.68%, while CNNs appeared in eight studies with accuracies between 70% and 95.40%. Although these ranges demonstrate generally strong performance, they also indicate that no single method consistently outperforms others across all scenarios. Differences in data types, preprocessing approaches, model architectures, and evaluation strategies preclude definitive conclusions regarding the best-performing AI approach. Furthermore, no studies directly compared model performance against clinicians, since clinician diagnosis typically served as the reference standard for labeling training and test data. Consequently, it remains unclear how these models perform relative to expert clinical judgment. For these reasons, a meta-analysis was not performed, and accuracy values should be interpreted within the context of each study’s design rather than through direct comparisons across models.

Future research should prioritize improved demographic reporting, larger datasets, and more consistent study methodologies. Coordinated multi-center efforts could establish shared protocols for collecting and reporting tremor data while enabling the development of large, publicly available datasets that allow fair comparison of AI models. These efforts should also prioritize tools that integrate into routine clinical practice. Nearly all included studies relied on device-based data such as accelerometers, EMG, and video recordings, whereas AI applications based solely on clinical history or physical examination data were notably absent. Given the practicality and lower resource demands of clinical data, exploring AI methods that incorporate or rely on clinical assessments represents an important future direction.

Although EMG provides detailed electrophysiological information, its routine clinical use can be limited by equipment requirements, time demands, and the need for specialized expertise [65,66]. In contrast, approaches such as Archimedes’ spirals, accelerometers, and gyroscopes are more accessible, particularly when integrated into smartphone-based platforms. One potential approach could involve a smartphone application developed collaboratively by movement disorder specialists and computer scientists. However, smartphone- and wearable-based assessments should currently be considered exploratory rather than validated clinical solutions. Although smartphones can capture accelerometer and gyroscope data, their size, weight, sensor placement, and the need for active holding may alter tremor biomechanics and signal characteristics, effects that wearable devices might mitigate. These factors have not been systematically studied and may introduce confounding effects. Consequently, both smartphone- and wearable-based approaches should currently be viewed as hypothesis-generating tools rather than ready-to-deploy clinical instruments.

### Limitations

Several limitations should be considered when interpreting this review. First, only English-language articles were included, which may have excluded relevant non-English studies. Second, studies comparing ET only to healthy control groups or primarily focusing on differentiating another tremor type (e.g., PD) were excluded. Although these studies contribute to the broader literature on ML-based tremor classification, the scope of this review was intentionally limited to work differentiating ET from at least one other tremor type. Additionally, most included studies relied on clinically well-characterized and relatively unambiguous cases from single centers. As a result, current ML models have largely been trained and evaluated in idealized settings, and their performance in diagnostically challenging or longitudinally evolving tremor cases remains uncertain. Addressing this gap will be essential for meaningful clinical translation.

The level of methodological and reporting detail varied considerably across studies. Some articles focused primarily on ML algorithm development, whereas others emphasized data collection and patient characteristics. This inconsistency limited the ability to calculate overall sensitivity and specificity across all 46 studies, thereby affecting generalizability. Similarly, the types of patient data used for model training differed widely, making it difficult to determine which data modalities are most effective for ML-based tremor classification. These limitations further emphasize the need for standardized data collection protocols and reporting guidelines to improve model development and evaluation. No included studies employed case-matched controls or randomized controlled trial designs. Our exclusion of studies comparing ET only to healthy controls likely filtered out some RCTs, contributing to the absence of such designs. Future work incorporating case-matched cohorts and randomized methodologies would strengthen evidence regarding AI performance.

Finally, because clinician diagnosis served as the reference standard in nearly all studies, model performance is inherently constrained by the accuracy and consistency of clinical tremor classification itself. Some patients labeled with ET may not truly have the condition, while others diagnosed with alternative tremor types may actually have ET. This diagnostic uncertainty introduces label noise, meaning that ML model performance largely reflects agreement with clinician judgment rather than identification of a definitive biological substrate. In addition, diagnostic adjudication methods were inconsistently reported. Few studies described whether diagnoses were established by single clinicians, expert panels, or consensus processes, and descriptions of research diagnostic criteria were often limited. This lack of transparency reduces confidence in the ground truth labels used for model training and highlights a critical gap in the literature. Moreover, few studies reported detailed error analyses examining the nature of misclassifications, such as cases where ML models labeled tremor as ET while clinicians diagnosed another tremor type, or vice versa. Given the imperfect ground truth based on clinician diagnosis, further work examining model failure modes will be essential. Consequently, reported accuracies should be interpreted as measures of concordance with expert classification rather than objective diagnostic truth. This may also help explain why average diagnostic accuracy has remained relatively stable over the past two decades despite advances in AI technology.

### Conclusion

In conclusion, this scoping review demonstrates that ML holds significant promise as a diagnostic aid for differentiating ET from other tremor types, a task that remains challenging due to overlapping clinical features and the absence of a definitive biomarker. Across 46 studies, a variety of ML models were trained using diverse data sources ranging from resource-intensive modalities such as EMG to more accessible inputs like smartphone-based accelerometry and gyroscope data. Although reported accuracies ranged from 60% to 100%, heterogeneity in data types, preprocessing methods, and model architectures limited direct comparison across studies. Nevertheless, these findings highlight that tremor classification is fundamentally a pattern recognition problem, an area in which ML is particularly well suited.

The clinical impact of these tools will depend on broader efforts to develop large, standardized, publicly available datasets, automate preprocessing pipelines, and prioritize clinically feasible data sources (Figure 2). Rather than functioning as standalone solutions, ML systems should be designed to complement the clinical reasoning of movement disorder specialists and neurologists, particularly in diagnostically ambiguous cases. International societies could play a key role in coordinating these efforts by forming multidisciplinary task forces that include both movement disorder neurologists and computer scientists. When effectively integrated, ML models may enhance pattern recognition beyond what is achievable through clinical observation alone, providing neurologists with an additional layer of decision support to improve diagnostic confidence.

## Financial Disclosures for the previous 12 months

KEH is supported by the National Science Foundation Research Traineeship (NRT) Program under Grant No. 2125872. AWA is a member of the faculty of the University of Colorado and is supported by university funds. She has served as a site investigator for studies sponsored by Michael J Fox Foundation for Parkinson’s Research, Parkinson Study Group, Aligning Science Across Parkinson’s (ASAP) Initiative, Koneksa Health, Biohaven Pharmaceuticals, Inc., Bial, and the NIH NINDS. She receives grant funding from NIH NICHD. She has received honoraria for consultancy to Aevum through Parkinson Study Group. She is a consultant for PhotoPharmics, Inc. The other authors declare that there are no additional disclosures to report.

## Additional Files

The additional files for this article can be found as follows:

10.5334/tohm.1182.s1 Electronic Supplementary Material Appendix S1. Systematic literature search conducted April 19, 2025.

10.5334/tohm.1182.s2 Electronic Supplementary Material Appendix S2. PICO Criteria defined for article inclusion.

10.5334/tohm.1182.s3 Electronic Supplementary Material Appendix S3. All studies included for article review with their study design.

10.5334/tohm.1182.s4 Electronic Supplementary Material Appendix S4. Complete bias assessment scores for each study.

10.5334/tohm.1182.s5 Electronic Supplementary Material Appendix S5. Patients from included studies divided into tremor types.

10.5334/tohm.1182.s6 Electronic Supplementary Material Appendix S6. Full list of patient data acquired by each article.

10.5334/tohm.1182.s7 Electronic Supplementary Material Appendix S7. Full list of AI models used by each article.
