# Supplementary material for: Machine Learning for Differentiating Essential Tremor: A Scoping Review
Source: Tremor Other Hyperkinet Mov (N Y). 2026 May 6;16:28. doi: 10.5334/tohm.1182 (PMC13155088; doi:10.5334/tohm.1182)
Supplement: Electronic Supplementary Material Appendix S1. — Systematic literature search conducted April 19, 2025. [file tohm-16-1-1182-s1.pdf]

**Electronic Supplementary Material Appendix S1**

Bibliographic Literature searching

| **Database** | **Hits** |
|---|---|
| 1. Scopus | 436 |
| 2. PubMed | 223 |
| 3. Cochrane Library | 62 |
| Total | 721 |
| Duplicates | 161 |
| **Unique studies to screen** | **560** |

Database: **Scopus**
Host: Elsevier
Data Parameters: none
Date Searched: 04/19/2025
Searcher: Joseph C. Melott Strategy Checked By: Adeel A. Memon, MD, PhD

Search Strategy:

| # | Searches | Results |
|---|---|---|
| 1 | (TITLE-ABS-KEY(essential AND tremor) | 9779 |
| 2 | (TITLE-ABS-KEY("artificial intelligence") | 674,667 |
| 3 | (TITLE-ABS-KEY("deep learning") | 551,552 |
| 4 | (TITLE-ABS-KEY("machine learning") | 836,527 |
| 5 | (TITLE-ABS-KEY("computer vision") | 256,187 |
| 6 | (TITLE-ABS-KEY("random forest") | 152,695 |
| 7 | (TITLE-ABS-KEY("XGBoost") | 30,444 |
| 8 | (TITLE-ABS-KEY("support vector machine") | 234,231 |
| 9 | (TITLE-ABS-KEY("K-nearest neighbor") | 64,211 |
| 10 | (TITLE-ABS-KEY("unsupervised learning") | 32,618 |
| 11 | (TITLE-ABS-KEY("supervised learning") | 88,315 |
| 12 | (TITLE-ABS-KEY("clustering") | 513,591 |
| 13 | (TITLE-ABS-KEY("dimensionality reduction") | 39,701 |
| 14 | (TITLE-ABS-KEY("association rule learning") | 300 |
| 1 | (TITLE-ABS-KEY("anomaly detection") | 51,92 |

| | | |
|---|---|---|
| 5 | | 4 |
| 16 | (TITLE-ABS-KEY(“generative models”) | 24,576 |
| 17 | (TITLE-ABS-KEY(“neural networks”) | 1,149,700 |
| 18 | (TITLE-ABS-KEY(“memory networks”) | 21,921 |
| 19 | (TITLE-ABS-KEY(“transformer models”) | 13,675 |
| 20 | (TITLE-ABS-KEY(“autoencoders”) | 12,860 |
| 21 | (TITLE-ABS-KEY(generative adversarial networks”) | 46,782 |
| 22 | #1 AND (#2 OR #3 OR #4 OR #5 OR #6 OR #7 OR #8 OR #9 OR #10 OR #11 OR #12 OR #13 OR #14 OR #15 OR #16 OR #17 OR #18 OR #19 OR #20 OR #21) | 436 |

Notes: Copy/paste search into the advanced query in scopus.com.

Database: **PubMed**
Host: National Library of Medicine
Data Parameters: none
Date Searched: 4/19/2025
Searcher: Joseph C. Melott Strategy Checked By: Adeel A. Memon, MD, PhD

Search Strategy:

| **#** | **Searches** | **Results** |
|---|---|---|
| 1 | "essential tremor"[MeSH Terms] OR ("essential"[All Fields] AND "tremor"[All Fields]) OR "essential tremor"[All Fields] | 6230 |
| 2 | "AI"[All Fields] OR ("artificial intelligence"[MeSH Terms] OR ("artificial"[All Fields] AND "intelligence"[All Fields]) OR "artificial intelligence"[All Fields]) OR ("machine learning"[MeSH Terms] OR ("machine"[All Fields] AND "learning"[All Fields]) OR "machine learning"[All Fields]) OR ("deep learning"[MeSH Terms] OR ("deep"[All Fields] AND "learning"[All Fields]) OR "deep learning"[All Fields]) OR "computer vision"[All Fields] OR "supervised learning"[All Fields] OR "random forest"[All Fields] OR "XGBoost"[All Fields] OR "support vector machine"[All Fields] OR "K-nearest neighbors"[All Fields] OR "unsupervised learning"[All Fields] OR "clustering"[All Fields] OR "dimensionality reduction"[All Fields] OR "association rule learning"[All Fields] OR "anomaly detection"[All Fields] OR "generative models"[All Fields] OR "neural networks"[All Fields] OR "memory networks"[All Fields] OR "transformer models"[All Fields] OR "autoencoders"[All Fields] | 1,157,526 |
| 3 | "artificial intelligence"[MeSH Terms] OR ("artificial"[All Fields] AND "intelligence"[All Fields]) OR "artificial intelligence"[All Fields | 296,726 |
| 4 | "machine learning"[MeSH Terms] OR ("machine"[All Fields] AND "learning"[All Fields]) OR "machine learning"[All Fields] | 196,259 |
| 5 | "deep learning"[MeSH Terms] OR ("deep"[All Fields] AND "learning"[All Fields]) OR "deep learning"[All Fields] | 102,229 |
| 6 | #1 AND (#2 OR #3 OR #4 OR #5) | 223 |

Notes: N/A

Database: **Cochrane Library**
Host: Wiley
Data Parameters: none
Date Searched: 4/19/2025
Searcher: Joseph C. Melott Strategy Checked By: Adeel A. Memon, MD, PhD

Search Strategy:

| # | Searches | Results |
|---|---|---|
| 1 | Essential tremor | 709 |
| 2 | Artificial Intelligence | 2981 |
| 3 | MeSH [artificial intelligence] explode all trees | 3427 |
| 4 | “machine learning” | 3361 |
| 5 | “deep learning” | 1281 |
| 6 | “computer vision” | 206 |
| 7 | “supervised learning” | 54 |
| 8 | “random forest” | 865 |
| 9 | “XGBoost” | 146 |
| 10 | “support vector machine” | 568 |
| 11 | “K-nearest machine” | 38 |
| 12 | “unsupervised learning” | 18 |
| 13 | “clustering” | 5524 |
| 14 | “dimensionality reduction” | 95 |
| 15 | “association rule learning” | 0 |
| 16 | “anomaly detection” | 16 |

| | | |
|---|---|---|
| 17 | “generative models” | 9 |
| 18 | “neural networks” | 1121 |
| 19 | “memory networks” | 38 |
| 20 | “transformer models” | 1 |
| 21 | “autoencoders” | 7 |
| 22 | #2 OR #3 OR #4 OR #5 OR #6 OR #7 OR #8 OR #9 OR #10 OR #11 OR #12 OR #13 OR #14 OR #15 OR #16 OR #17 OR #18 OR #19 OR #20 OR #21 | 14922 |
| 23 | #1 AND #22 | 68 |

Notes: Built search using Advanced Search->Search Manager to enter subject headings and keyword terms and combining the concepts using line-by-line formatting.
Dataset saved as: citation-export.ris
