# Supplementary material for: Machine Learning for Differentiating Essential Tremor: A Scoping Review
Source: Tremor Other Hyperkinet Mov (N Y). 2026 May 6;16:28. doi: 10.5334/tohm.1182 (PMC13155088; doi:10.5334/tohm.1182)
Supplement: Electronic Supplementary Material Appendix S2. — PICO Criteria defined for article inclusion. [file tohm-16-1-1182-s2.pdf]

| **PICO** | **Criteria** |
|---|---|
| P - *Population* | Patients with tremor undergoing diagnostic evaluation to distinguish essential tremor (ET) from other tremor types |
| I - *Intervention* | Artificial intelligence (AI) or machine learning (ML) methods used to differentiate ET from other tremor disorders |
| C - *Comparator* | Diagnosis made by clinicians (e.g., neurologists or movement disorder specialists) or other standard diagnostic tools |
| O - *Outcome* | Diagnostic performance metrics of AI methods (e.g., accuracy, sensitivity, specificity, AUC) |
| S - *Study Characteristics* | Any study type excluding systematic reviews/meta analyses, animal studies, studies not written in english |
