# Supplementary material for: Machine Learning for Differentiating Essential Tremor: A Scoping Review
Source: Tremor Other Hyperkinet Mov (N Y). 2026 May 6;16:28. doi: 10.5334/tohm.1182 (PMC13155088; doi:10.5334/tohm.1182)
Supplement: Electronic Supplementary Material Appendix S3. — All studies included for article review with their study design. [file tohm-16-1-1182-s3.pdf]

**Authors**	**Publication Year**	**Study Type**
Adeshnia et al.	2024	Diagnostic Test Accuracy Study
Ai et al.	2007	Diagnostic Test Accuracy Study
Ai et al.	2008	Diagnostic Test Accuracy Study
Ai et al.	2011	Diagnostic Test Accuracy Study
Anandapadmanabhan et al.	2025	Diagnostic Test Accuracy Study
Aracri et al.	2024	Diagnostic Test Accuracy Study
Aubin et al.	2012	Diagnostic Test Accuracy Study
Balachandar et al.	2022	Diagnostic Test Accuracy Study
Chandra Reddy et al.	2024	Diagnostic Test Accuracy Study
Darnall et al.	2012	Diagnostic Test Accuracy Study
Duque et al.	2020	Diagnostic Test Accuracy Study
Engin et al.	2007	Diagnostic Test Accuracy Study
Ferreira et al.	2022	Diagnostic Test Accuracy Study
Ghassemi et al.	2016	Diagnostic Test Accuracy Study
Gonzalez et al.	2014	Diagnostic Test Accuracy Study
Groznik et al.	2013	Diagnostic Test Accuracy Study
Hossen et al.	2012	Diagnostic Test Accuracy

		Study
Hossen et al.	2022	Diagnostic Test Accuracy Study
Ishii et al.	2020	Diagnostic Test Accuracy Study
Jakubowski et al.	2002	Diagnostic Test Accuracy Study
Kovalenko et al.	2021	Diagnostic Test Accuracy Study
Lee et al.	2023	Diagnostic Test Accuracy Study
Li et al.	2023	Diagnostic Test Accuracy Study
Lin et al.	2023	Diagnostic Test Accuracy Study
Locatelli et al.	2020	Diagnostic Test Accuracy Study
Moon et al.	2020	Diagnostic Test Accuracy Study
Nanayakkara et al.	2025	Diagnostic Test Accuracy Study
Nanda et al.	2015	Diagnostic Test Accuracy Study
Oktay et al.	2020	Diagnostic Test Accuracy Study
Piepjohn et al.	2022	Diagnostic Test Accuracy Study
Ranjan et al.	2020	Diagnostic Test Accuracy Study
Saad et al.	2024	Diagnostic Test Accuracy Study
Sanderson et al.	2020	Diagnostic Test Accuracy Study
Seedat et al.	2020	Diagnostic Test Accuracy Study

Shahtalebi et al.	2020	Diagnostic Test Accuracy Study
Shahtalebi et al.	2021	Diagnostic Test Accuracy Study
Skaramagkas et al.	2020	Diagnostic Test Accuracy Study
Skaramagkas et al.	2021	Diagnostic Test Accuracy Study
Spyers-Ashby et al.	1999	Diagnostic Test Accuracy Study
Surangsrirat et al.	2016	Diagnostic Test Accuracy Study
Tang et al.	2024	Diagnostic Test Accuracy Study
Tavakkoli et al.	2014	Diagnostic Test Accuracy Study
Teo et al.	2024	Diagnostic Test Accuracy Study
Vescio et al.	2023	Diagnostic Test Accuracy Study
Weede et al.	2024	Diagnostic Test Accuracy Study
Xing et al.	2022	Diagnostic Test Accuracy Study
Yang et al.	20220	Diagnostic Test Accuracy Study
