# Supplementary material for: Machine Learning for Differentiating Essential Tremor: A Scoping Review
Source: Tremor Other Hyperkinet Mov (N Y). 2026 May 6;16:28. doi: 10.5334/tohm.1182 (PMC13155088; doi:10.5334/tohm.1182)
Supplement: Electronic Supplementary Material Appendix S4. — Complete bias assessment scores for each study. [file tohm-16-1-1182-s4.pdf]

Bias assessment of **Diagnostic Test Accuracy Studies** using JBI critical appraisal checklist												
Study	Q1	Q2	Q3	Q4	Q5	Q6	Q7	Q8	Q9	Q10	Score	Score %
**Adeshina et al. 2024**	N	Y	N	N	Y	N	N	Y	Y	Y	5	50%
**Ai et al. 2007**	N	Y	N	N	N	N	N	Y	Y	Y	4	40%
**Ai et al. 2008**	N	Y	N	N	Y	N	N	Y	Y	Y	5	50%
**Ai et al. 2011**	N	Y	N	N	Y	N	N	Y	Y	Y	5	50%
**Anandapadmanabhan et al. 2025**	Y	Y	Y	Y	Y	Y	Y	Y	Y	Y	10	100%
**Aracri et al. 2024**	Y	Y	Y	Y	Y	Y	Y	Y	Y	Y	10	100%
**Aubin et al. 2012**	N	Y	Y	N	Y	N	N	Y	Y	Y	6	60%
**Balachandar et al. 2022**	Y	Y	Y	Y	Y	Y	Y	Y	Y	Y	10	100%
**Chandra Reddy et al. 2024**	N	Y	Y	N	Y	N	N	Y	Y	Y	6	60%
**Darnall et al. 2012**	N	Y	Y	N	N	Y	N	Y	Y	Y	6	60%
**Duque et al. 2022**	N	Y	Y	N	Y	N	N	Y	Y	Y	6	60%
**Engin et al. 2007**	N	Y	N	N	N	N	N	Y	Y	Y	4	40%
**Ferreira et al. 2022**	N	Y	Y	Y	Y	Y	Y	Y	Y	Y	9	90%
**Ghassemi et al. 2016**	N	N	Y	Y	N	Y	Y	Y	Y	Y	7	70%
**Gonzalez et al. 2014**	N	Y	Y	N	N	N	N	Y	Y	Y	5	50%
**Groznik et al. 2013**	N	Y	Y	N	Y	Y	N	Y	Y	Y	7	70%
**Hossen et al. 2012**	N	Y	Y	N	Y	Y	N	Y	Y	Y	7	70%
**Hossen et al. 2022**	N	Y	Y	Y	Y	Y	Y	Y	Y	Y	9	90%
**Ishii et al. 2020**	N	Y	Y	Y	Y	Y	Y	Y	Y	Y	9	90%
**Jakubowski et al. 2002**	N	Y	Y	N	N	N	N	Y	Y	Y	5	50%
**Kovalenko et al. 202**	Y	Y	Y	Y	Y	Y	Y	Y	Y	Y	10	100%
**Lee et al. 2023**	N	Y	Y	Y	Y	Y	Y	Y	Y	Y	9	90%
**Li et al. 2023**	Y	Y	Y	Y	Y	Y	Y	Y	Y	Y	10	100%
**Lin et al. 2023**	Y	Y	Y	Y	Y	Y	Y	Y	Y	Y	10	100%
**Locatelli et al. 2020**	Y	Y	Y	Y	Y	Y	Y	Y	Y	Y	10	100%
**Moon et al. 2020**	Y	Y	Y	Y	Y	Y	Y	Y	Y	Y	10	100%
**Nanayakkara et al. 2025**	Y	Y	Y	Y	Y	Y	Y	Y	Y	Y	10	100%
**Nanda et al. 2015**	N	Y	Y	N	Y	N	N	Y	Y	Y	6	60%
**Oktay et al. 2020**	Y	Y	Y	Y	Y	Y	Y	Y	Y	Y	10	100%
**Piepjohn et al. 2022**	Y	Y	Y	Y	Y	Y	Y	Y	Y	Y	10	100%
**Ranjan et al. 2020**	N	Y	Y	N	N	N	N	Y	Y	Y	5	50%
**Saad et al. 2024**	N	Y	Y	Y	Y	Y	Y	Y	Y	Y	9	90%
**Sanderson 2020**	Y	Y	Y	Y	Y	Y	Y	Y	Y	Y	10	100%
**Seedat 2020**	N	Y	Y	Y	Y	N	Y	Y	Y	Y	8	80%
**Shahtalebi 2020**	Y	Y	Y	Y	Y	Y	Y	Y	Y	Y	10	100%
**Shahtalebi 2021**	Y	Y	Y	Y	Y	Y	Y	Y	Y	Y	10	100%
**Skaramagkas 2020**	Y	Y	Y	Y	Y	Y	Y	Y	Y	Y	10	100%
**Skaramagkas 2021**	Y	Y	Y	Y	Y	Y	Y	Y	Y	Y	10	100%
**Spyers-Ashby 1999**	Y	Y	Y	Y	Y	Y	Y	Y	Y	Y	10	100%
**Surangsrirat 2016**	Y	Y	Y	Y	Y	Y	Y	Y	Y	Y	10	100%
**Tang 2024**	Y	Y	Y	Y	Y	Y	Y	Y	Y	Y	10	100%
**Tavakkoli 2014**	Y	Y	Y	Y	Y	Y	Y	Y	Y	Y	10	100%
**Teo 2024**	N	Y	Y	Y	Y	Y	Y	Y	Y	Y	9	90%
**Vescio 2023**	Y	Y	Y	Y	Y	Y	Y	Y	Y	Y	10	100%
**Weede 2024**	Y	Y	Y	Y	Y	Y	Y	Y	Y	Y	10	100%
**Xing 2022**	Y	Y	Y	Y	Y	Y	Y	Y	Y	Y	10	100%
**Yang 2020**	Y	Y	Y	Y	Y	Y	Y	Y	Y	Y	10	100%
Average Score												**83.1915%**

Y: Yes N: No U: Unclear N/A: Not Applicable

Q1: Was a consecutive or random sample of patients enrolled?

Q2: Was a case control design avoided?
Q3: Did the study avoid inappropriate exclusions?
Q4: Were the index test results interpreted without knowledge of the results of the reference standard?
Q5: If a threshold was used, was it pre-specified?
Q6: Is the reference standard likely to correctly classify the target condition?
Q7: Were the reference standard results interpreted without knowledge of the results of the index test?
Q8: Was there an appropriate interval between index test and reference standard?
Q9: Did all patients receive the same reference standard?
Q10: Were all patients included in the analysis?
