# Supplementary material for: Machine Learning for Differentiating Essential Tremor: A Scoping Review
Source: Tremor Other Hyperkinet Mov (N Y). 2026 May 6;16:28. doi: 10.5334/tohm.1182 (PMC13155088; doi:10.5334/tohm.1182)
Supplement: Electronic Supplementary Material Appendix S5. — Patients from included studies divided into tremor types. [file tohm-16-1-1182-s5.pdf]

Electronic Supplementary Material Appendix S5. Patients from included studies divided into tremor types.

Tremor Type	Number of Patients	Articles Referenced
Essential Tremor	2358	ALL
Parkinson's Disease	2804	ALL except Ishii 2020
Physiologic Tremor	125	Ai 2007, 2008; Jakubowski 2002
Dystonic Tremor	391	Anandapadmanabhan 2025; Balachandar 2022
Cerebellar Tremor	7	Chandra Reddy 2024
Parkinson's Disease/Essential Tremor (Mixed Tremor)	35	Darnall 2012; Groznik 2013
Essential Tremor Plus	208	Anandapadmanabhan 2025
Cerebellar Ataxia	104	Anandapadmanabhan 2025; Ishii 2020
Multiple Sclerosis	17	Spyers-Ashby 1999
Other	2	Kovalenko 2021
**Total Number of Patients**	6051	
