# Supplementary material for: Machine Learning for Differentiating Essential Tremor: A Scoping Review
Source: Tremor Other Hyperkinet Mov (N Y). 2026 May 6;16:28. doi: 10.5334/tohm.1182 (PMC13155088; doi:10.5334/tohm.1182)
Supplement: Electronic Supplementary Material Appendix S6. — Full list of patient data acquired by each article. [file tohm-16-1-1182-s6.pdf]

Electronic Supplementary Material Appendix S6. Full list of patient data acquired by each article.

Patient Data Recorded	Number of Articles	Articles Referenced
9-Degrees of Freedom	4	Aubin 2012; Lin 2023; Sanderson 2020; Vescio 2023
Accelerometer	26	Ai 2007, 2008, 2011; Balachandar 2022; Engin 2007; Ferreira 2022; Ghassemi 2016; Gonzalez 2014; Hossen 2012, 2022; Jakubowski 2002; Li 2023; Locatelli 2020; Moon 2020; Nanayakkara 2025; Nanda 2015; Piepjohn 2022; Ranjan 2020; Shahtalebi 2020, 2021; Skaramagkas 2020, 2021; Spyers-Ashby 1999; Tang 2024; Weede 2024; Xing 2022
Archimedes Spirals	6	Anandapadmanabhan 2024; Darnall 2012; Groznik 2013; Ishii 2020; Seedat 2020; Yang 2020
Electromyography	9	Arcari 2024; Ghassemi 2016; Hossen 2012; Hossen 2022; Nanda 2015; Piepjohn 2022; Tang 2024; Tavakkoli 2014; Xing 2022
Gyroscope	5	Darnall 2012; Duque 2020; Ferreira 2022; Moon 2020; Surangsrirat 2016
Tasks (Drinking, Timed Up and Go)	2	Lin 2023; Teo 2024
Tremor Rating Scale	3	Balachandar 2022; Darnall 2012; Lee 2023
Video Recordings	5	Chandra Reddy 2024; Kovalenko 2021; Lee 2023; Oktay 2020; Saad 2024
**Method of Collection**		
Continuous Wearables	3	Lin 2023; Moon 2020; Vescio 2023
Smartphone	2	Balachandar 2022; Duque 2020
