# Supplementary material for: Machine Learning for Differentiating Essential Tremor: A Scoping Review
Source: Tremor Other Hyperkinet Mov (N Y). 2026 May 6;16:28. doi: 10.5334/tohm.1182 (PMC13155088; doi:10.5334/tohm.1182)
Supplement: Electronic Supplementary Material Appendix S7. — Full list of AI models used by each article. [file tohm-16-1-1182-s7.pdf]

**Name**	**Type**	**Number of Articles**	**Articles Referenced**
Artificial Neural Network	Supervised Learning	1	Engin 2007
Back Propagation Neural Network		4	Ai 2007, 2008; Hossen 2012; Xing 2022
Convolutional Neural Network		8	Ananthapadmanabhan 2025; Chandra Reddy 2024; Ishii 2020; Piepjohn 2022; Seedat 2020; Tavakkoli 2014; Weede 2024; Xing 2022
Cross Attention Mechanism Network		1	Tang 2024
Decision Tree		7	Darnall 2012; Ferreira 2022; Locatelli 2020; Moon 2020; Piepjohn 2022; Skaramagkas 2020; Skaramagkas 2021
Discriminant Analysis (Linear/Quadratic)		4	Locatelli 2020; Shahtalebi 2021; Skaramagkas 2020; Skaramagkas 2021
Ensemble Learning		2	Skaramagkas 2020; Skaramagkas 2021
Feed Forward Back Propagation Neural Network		2	Hossen 2022; Nanda 2015
Gaussian Process Classifier		1	Kovalenko 2021
Generalized Regression Neural Network		1	Yang 2020
Gradient Boosting/Extreme Gradient Boosting		6	Arcari 2024; Kovalenko 2021; Moon 2020; Saad 2024; Vescio 2023; Xing 2022

k Nearest Neighbors		9	Darnall 2012; Duque 2020; Ferreira 2022; Locatelli 2020; Moon 2020; Ranjan 2020; Skaramagkas 2020; Skaramagkas 2021; Spyers-Ashby 1999
Logistic Regression		5	Adeshina 2024; Kovalenko 2021; Lin 2023; Moon 2020; Xing 2022
Long Short Term Memory		4	Lee 2023; Nanayakkara 2025; Oktay 2020; Teo 2024
Multilayer Perceptron		3	Darnall 2012; Gonzalez 2014; Jakubowski 2002
Naive Bayes		3	Darnall 2012; Ferreira 2022; Locatelli 2020
Random Forest		7	Darnall 2012; Ferreira 2022; Kovalenko 2021; Moon 2020; Ranjan 2020; Vescio 2023; Xing 2022
Ridge Regression for Classification		1	Xing 2022
Support Vector Machine		18	Ai 2011; Aubin 2012; Darnall 2012; Duque 2020; Ferreira 2022; Ghassemi 2016; Kovalenko 2021; Li 2023; Locatelli 2020; Moon 2020; Ranjan 2020; Saad 2024; Sanderson 2020; Skaramagkas 2020; Skaramagkas 2021; Surangsrirat 2016; Tavakkoli 2014; Xing 2022
Gaussian Mixture Model	Unsupervised Learning	1	Ranjan 2020
k Means		1	Ranjan 2020

Patch-wise Hierarchical Transformer Network (PHTnet)	Semi-supervised Learning	1	Shahtalebi 2020
Uniform Manifold Approximation and Projection for Dimension Reduction (UMAP)		1	Balachandar 2022
Argument-Based Machine Learning	Multiple	1	Groznik 2013
Leave One Out Cross Validation	Validation	3	Balachandar; Li 2023; Lin 2023
